# Extended-Spectrum β-Lactamase-Producing *Escherichia coli* and Pediatric UTIs: A Review of the Literature and Selected Experimental Observations

**DOI:** 10.3390/antibiotics14121284

**Published:** 2025-12-18

**Authors:** Vanessa Tamas, Erlinda R. Ulloa, Monika Kumaraswamy, Samira Dahesh, Raymond Zurich, Victor Nizet, Alison Coady

**Affiliations:** 1 Department of Pediatrics, University of California San Diego School of Medicine, La Jolla, CA 92093, USA; 2 Department of Pediatrics, University of California Irvine School of Medicine, Irvine, CA 92697, USA; chulie.ulloa@uci.edu; 3 Division of Infectious Disease, Children’s Hospital of Orange County, Orange, CA 92868, USA; 4 Department of Medicine, Division of Infectious Diseases, University of Texas Tyler, Tyler, TX 75799, USA; monika.kumaraswamy@uttyler.edu; 5 Department of Cellular & Molecular Biology, University of Texas Tyler, Tyler, TX 75799, USA; 6 Division of Host-Microbe Systems and Therapeutics, Center for Immunity, Infection and Inflammation, University of California San Diego School of Medicine, La Jolla, CA 92093, USA; sdahesh@health.ucsd.edu (S.D.); rhzurich@ucdavis.edu (R.Z.); vnizet@health.ucsd.edu (V.N.); 7 Department of Microbiology and Immunology, The University of Texas Medical Branch, Galveston, TX 77555, USA

**Keywords:** *Escherichia coli*, urinary tract infection, antimicrobial resistance, pediatric infection

## Abstract

Urinary tract infections (UTIs) are common in children and are predominantly caused by uropathogenic *Escherichia coli* (UPEC). An increasing proportion of these strains produce extended-spectrum β-lactamases (ESBLs), which render β-lactam antibiotics ineffective. Interestingly, some patients with ESBL-producing UTIs improve clinically following treatment with antibiotics like cephalexin, despite demonstrated in vitro resistance. Working alongside and at times synergistically with antibiotics, host immune factors, such as the antimicrobial peptide cathelicidin (LL-37), contribute to bacterial clearance through direct killing and inhibition of biofilm formation. In this review, we summarize the current understanding of pediatric ESBL-producing UPEC infections and present selected in vitro and in vivo experimental data evaluating the combined effects of LL-37 and cephalexin on clinical isolates. Although no synergy was observed, ESBL-producing isolates demonstrated reduced bacterial burden in vivo compared to a non-ESBL UPEC strain. These findings suggest that host immune factors and environmental conditions may influence the fitness and virulence of drug-resistant UTI pathogens, warranting further investigation.

## 1. Introduction

Urinary tract infections (UTIs) are among the most common bacterial infections in children and represent a substantial clinical burden. The rise in uropathogenic *Escherichia coli* (UPEC) strains that produce extended-spectrum β-lactamases (ESBLs) complicates UTI management, as these enzymes hydrolyze β-lactam antibiotics, rendering them ineffective. Prevalence varies widely, with certain locations reporting over 40%; recent studies report a national prevalence of 3.5% [1,2]. Paradoxically, some patients with ESBL-producing UTIs respond favorably to antibiotics such as cephalexin, which are predicted to be ineffective based on traditional in vitro susceptibility testing. This discrepancy suggests that additional factors—particularly host immune responses—may influence clinical outcomes in ways not captured by conventional testing.

In this review, we discuss the pathogenesis, treatment, and emerging challenges posed by ESBL-producing UPEC in pediatric UTIs. We will also highlight the role of host immune factors, such as the antimicrobial peptide cathelicidin (LL-37), in UTI defense. To explore the potential interplay between LL-37 and cephalexin, we conducted a series of in vitro and in vivo experiments using clinical ESBL UPEC isolates. Our goal is to better understand how host–pathogen interactions and immune-mediated mechanisms may contribute to clinical improvement despite discordant antibiotic therapy.

## 2. Epidemiology of Pediatric Urinary Tract Infections

UTIs represent a significant healthcare burden in pediatrics, accounting for 5–14% of all pediatric emergency department (ED) visits, approximately 1.5 million annual ambulatory care encounters, and 50,000 hospital admissions annually [3,4]. The prevalence of UTIs varies according to age, sex, and circumcision status. Among infants ≤60 days of age, incidence is higher in uncircumcised males (1.42 per 100 patient-years) compared to circumcised males (0.49) and females (0.94) [5]. This increased risk is attributed to bacterial colonization of the foreskin and physiologic phimosis, which retains uropathogens near the urethral meatus.

Beyond infancy, females account for the majority of pediatric UTI cases, primarily due to anatomical factors such as a shorter urethra, which facilitates ascending infection. Additional risk factors include congenital abnormalities of the kidney and urinary tract (CAKUT) and neurologic conditions that impair voiding. These conditions predispose to urinary stasis and incomplete bladder emptying, creating an environment favorable to bacterial replication, adherence, and invasion [4]. Among children with vesicourethral reflex (VUR), the risk of recurrent febrile UTIs is particularly elevated, often requiring prophylaxis and surveillance. A recent multicenter study of infants with CAKUT in Taiwan confirmed that structural abnormalities are independently associated with both first and recurrent UTIs, even after adjusting for age and sex [6].

In community-acquired UTIs, *E. coli* remains the predominant uropathogen, accounting for 83% of UTIs in females and 50% in males [4,7]. Other commonly isolated organisms include *Enterococcus* spp., *Proteus mirabilis*, *Klebsiella* spp., *Pseudomonas aeruginosa*, and *Enterobacter* spp. [7]. In neonates and young infants, especially those with immature immune defenses, non-UPEC pathogens like group B *Streptococcus*, *Staphylococcus aureus*, and coagulase-negative staphylococci are also encountered, highlighting a broader spectrum of potential pathogens during early life [8,9,10].

## 3. Treatment of Bacterial Urinary Tract Infections

Empiric antibiotic therapy is central to UTI management in children and is initiated while awaiting final culture and susceptibility results. Cephalexin, a first-generation cephalosporin, is often preferred due to its narrow antimicrobial spectrum, favorable pharmacokinetics, and high oral bioavailability [7]. It is well absorbed and is excreted unchanged in the urine within 6 h of administration, making it effective for lower urinary tract infections [11].

The American Academy of Pediatrics (AAP) recommends cephalexin, amoxicillin-clavulanic acid, trimethoprim-sulfamethoxazole (TMP-SMX), and select oral third generation cephalosporins (cefdinir, cefixime, and cefpodoxime) for initial therapy in febrile infants and young children with uncomplicated UTI [12]. Amoxicillin alone is generally avoided due to its widespread resistance among UPEC isolates. Ciprofloxacin may be considered for UTIs caused by highly resistant organisms, including ESBL producers, although concerns about arthropathy limits its use in children. Nitrofurantoin is suitable for uncomplicated cystitis but is not recommended for pyelonephritis due to its limited tissue penetration.

The selection of empiric therapy should be informed by local susceptibility patterns and any prior culture results. Once susceptibility data become available, treatment should narrow accordingly. According to the Infectious Diseases Society of America (IDSA), nitrofurantoin and TMP-SMX are preferred agents for uncomplicated cystitis caused by ESBL-Enterobacterales (ESBL-E), which includes *E. coli* [13]. Fluoroquinolones and carbapenems may be used as alternatives when susceptibility is confirmed, though carbapenems are typically reserved for complicated or invasive infections. Oral fosfomycin, although not widely used in pediatrics, is an additional option, for *E. coli* mediated cystitis. For more complicated infections such as pyelonephritis, TMP-SMX, ciprofloxacin, or levofloxacin are preferred if active. Otherwise, intravenous carbapenems or aminoglycosides can be considered, balancing efficacy with potential toxicities [13].

## 4. Antibiotic Resistance Trends with an Emphasis on *Escherichia coli*

An increasing number of Gram-negative pathogens now produce β-lactamases—enzymes that inactivate β-lactam antibiotics by hydrolyzing the β-lactam ring. These plasmid-mediated enzymes are diverse and rapidly evolving, with over 200 distinct variants described to date [14]. ESBLs are a subset of β-lactamases that hydrolyze first-, second-, and third-generation cephalosporins, although they do not affect carbapenems, which remain stable against ESBL-mediated hydrolysis [15]. ESBL-producing *E. coli* were first reported in hospitalized adults and long-term care facility residents in the 1980s. Since then, these resistant strains have disseminated globally, now causing both hospital- and community- acquired UTIs in infants and children. A dominant lineage contributing to this spread is *E. coli* ST131, which harbors CTX-M-type ESBLs and exhibits high virulence and multidrug resistance [16]. The reported prevalence of pediatric UTIs caused by ESBL-producing *E. coli* varies widely from 1% to over 40%, with higher reported rates among children in the Middle East and Indian Subcontinent [17,18,19,20,21,22,23].

Several risk factors are associated with ESBL UTI acquisition in children including underlying urologic abnormalities, renal disease, recent hospitalization, and prior antibiotic exposure—especially prophylactic antibiotics in children with recurrent infections [24,25,26]. In the United States, ESBL-producing *E. coli* accounted for approximately 3% of pediatric UTI isolates in 2010, with the highest prevalence among children aged 1–5. Recent surveillance data suggest that the rate continues to rise slowly, exceeding 3.5% in some settings [27,28].

As ESBL-producing pathogens become more common, a growing number of children discharged from the ED with UTIs are prescribed empiric antibiotics to which their infecting organisms are later found to be resistant. This scenario, known as discordant therapy, has traditionally raised concern. However, several studies report favorable clinical and microbiologic outcomes even when discordant antibiotic therapy is administered (Table 1, [29,30,31,32,33,34,35]). For example, a retrospective study of 45 infants with ESBL UTIs at a pediatric ED in San Diego, found that none required readmission or escalation of care during treatment [35]. In a larger retrospective study of 316 children, only 2.2% required a repeat ED visit or intensive care unit (ICU) admission while on discordant therapy [34]. Among patients with repeat cultures, 84% had resolution of pyuria, and 65% had sterile follow-up cultures while on discordant therapy [34]. While some cases may have resolved spontaneously, these studies challenge the assumption that in vitro resistance always predicts clinical failure

## 5. UTI Pathogenesis

Despite the near-constant exposure of the uroepithelium to microbes, UTIs remain relatively uncommon, reflecting the strength of local host defense mechanisms. A UTI typically begins with bacterial ascension through the urethra, followed by adherence to bladder uroepithelium via pili, and subsequent formation of intracellular bacterial communities (IBCs). These IBCs arise when a single bacterium invades a uroepithelial cell and replicates intracellularly, forming communities of up to 10^6^ bacteria [36]. Functionally analogous to biofilms, IBCs promote bacterial persistence and may contribute to progression from cystitis to pyelonephritis if left untreated. Key uroepithelial defenses that help maintain sterility include efficient bladder emptying, high velocity urine flow, shifts in urine osmolarity and pH, the secretion of soluble IgA and continuous production of Tamm-Horsfall Protein, which disrupts bacterial adhesion [37,38,39]. These barriers are further reinforced by the production of antimicrobial peptides (AMPs) such as α and β-defensins, RNase 7, and cathelicidin (LL-37), which are rapidly mobilized in response to uropathogen invasion [40,41,42,43,44]. AMPs bind to negatively charged bacterial membranes, disrupt membrane integrity, induce pore formation, and dysregulate ion gradients— ultimately leading to bacterial death [45]. Uropathogenic bacteria must overcome these highly developed and multifaceted defensive mechanisms to adhere to the uroepithelia, replicate, and cause a UTI.

## 6. Cathelicidin in the Urinary Tract

The antimicrobial peptide cathelicidin is a critical effector in mucosal defense. Produced by epithelial and immune cells, expression is induced by multiple stimuli, such as vitamin D in humans via VDR [46], microbial products via TLR signaling [47,48], and pro-inflammatory mediators [49,50]. Neutrophils are the primary source of cathelicidin, storing the inactive precursor (hCAP18) in secondary granules [51]. Upon secretion, cell- and tissue-specific proteases, such as kallikrein and proteinase-3, cleave the precursor into its active peptide LL-37 [52,53]. LL-37 is a cationic, amphipathic, α-helical peptide with potent killing activity against bacteria, fungi, viruses, and parasites [54,55,56,57,58,59,60,61]. Its strong positive net charge promotes electrostatic binding to negatively charged bacterial surface components, such as LPS and lipid membranes, and its amphipathic helical structure facilitates insertion into lipid bilayers, leading to membrane disruption and permeabilization [62,63]. Following permeabilization, LL-37 influx into bacterial cytoplasm leads to a destructive non-specific aggregation of DNA and ribosomes that interferes with essential cellular processes [64,65,66]. Collectively, LL-37 interaction causes cell wall and membrane deformation, breakdown of transmembrane potential, cytoplasmic disorganization, macromolecular aggregation, and eventual cell death [64,65,66,67]. In addition to direct killing activity, LL-37 prevents and disrupts biofilm formation by inhibiting substrate attachment, extracellular matrix formation, and quorum-sensing [68,69,70,71,72].

LL-37 also interacts with host cells to modulate a diverse range of immune responses. It mediates signaling via multiple receptors, including G-protein coupled receptors (GPCRs) MrgX2, FPR2, and CXCR2, and the purinergic receptor P2X7 [73,74,75]. These interactions enable LL-37 to mediate processes such as chemotaxis, the production of inflammatory mediators, cellular activation, differentiation, and proliferation across many cell types, including macrophages, neutrophils, NK cells, T cells, mast cells, eosinophils and epithelial cells [73,74,75,76,77,78,79,80,81,82,83,84,85]. Due to its positive charge (+6), LL-37 forms complexes with nucleic acids, such as mitochondrial DNA (mtDNA), genomic DNA in neutrophil extracellular traps (NETs), and RNA [86,87,88,89]. These LL-37:nucleic acid complexes activate toll-like receptor (TLR) signaling, amplifying the Type I interferon response and promoting inflammation. Additionally, LL-37 is a critical structural and functional component of NETs, which enable neutrophils to trap and kill microbes independently of phagocytosis.

In the urinary tract, cathelicidin is constitutively expressed by kidney proximal tubular cells and bladder epithelial cells, with its expression further increased by infiltrating leukocytes during infection [40]. UPEC exposure triggers rapid upregulation of cathelicidin in renal tissue within minutes, and elevated levels of cathelicidin are detected in both urine and plasma of patients with active UTIs [40,45,90]. The susceptibility of bacterial strains to cathelicidin correlates with clinical disease severity. UPEC strains causing pyelonephritis exhibit greater resistance to LL-37 compared to those causing cystitis [40,45]. Similarly, fecal *E. coli* strains from patients with recurrent UTIs are more resistant to cathelicidin than strains from healthy individuals, highlighting its potential role in shaping disease recurrence [45]. LL-37 also disrupts curli fimbriae polymerization, a process crucial for UPEC adherence to bladder epithelial cells, thus inhibiting biofilm and IBC formation [69]. Beyond its direct antimicrobial role, cathelicidin also acts as a chemoattractant to recruit and activate neutrophils and other innate immune cells to the urinary tract. LL-37 instilled into mouse bladders induces bladder inflammation [91] and mice lacking cathelicidin exhibit reduced inflammation during UPEC infection [92]. However, mice deficient in cathelicidin exhibit increased bacterial adherence to the uroepithelium and disease severity in some models [40], though not in others [92], underscoring its complex role in both antimicrobial and immune activity.

Both its antimicrobial function and diverse immune activity position LL-37 as a key player in maintaining immune homeostasis. It has been associated with appropriate wound healing, maintaining epithelial barrier function, and antitumor activity. Conversely, the aberrant production and function of LL-37 has also been implicated in driving inflammatory diseases, such as psoriasis, atherosclerosis, rosacea, rheumatoid arthritis, pancreatitis, and multiple sclerosis [93,94,95,96,97]. In addition to its antimicrobial and immunomodulatory roles, cathelicidin may directly modulate epithelial repair and barrier function after infection. LL-37 stimulates wound closure pathways and enhance epithelial restitution through EGFR transactivation and MAPK signaling in mucosal tissues [98,99,100]. While not yet studied in the bladder epithelium, these findings raise the possibility that cathelicidin contributes to mucosal healing post-infection, which may influence UTI recurrence risk and recovery.

## 7. Innate Immunity and Antibiotic Synergy

Multiple mechanisms underlie how antibiotics and the host immune system interact to control infection. Certain antibiotics modulate bacterial virulence factor expression, which in turn may influence host–pathogen dynamics [101,102]. Others exert direct immunomodulatory effects, altering immune cell recruitment, phagocytic function, or cytokine production [103,104].

Subinhibitory concentrations of β-lactam antibiotics can sensitize bacteria to killing by antimicrobial peptides. β-lactams irreversibly bind penicillin binding proteins (PBPs), transpeptidases that are essential for peptidoglycan synthesis in bacterial cell walls. Many pathogens, such as methicillin-resistant *Staphylococcus aureus* (MRSA), acquire altered PBPs that are less sensitive to inhibition by β-lactam antibiotics [105]. Other bacteria, like ESBL-producing *E. coli*, produce β-lactamases that degrade β-lactam antibiotics before they interact with PBPs. However, sub-inhibitory β-lactam exposure can still influence bacterial cell morphology, division, wall integrity, surface charge, and expression of virulence factors [102,106], indirectly affecting sensitivity to antibiotics. In MRSA, nafcillin exposure enhances susceptibility to cathelicidins and increases opsonophagocytosis by neutrophils [107]. Moreover, combinations of β-lactams and daptomycin show enhanced bactericidal activity and reduced resistance emergence in *S. aureus* models [106,108,109]. Other antibiotics, including azithromycin, demonstrate synergy with cathelicidin against several multidrug-resistant Gram-negative pathogens including *P. aeruginosa*, *K. pneumoniae*, and *A. baumannii* [110,111,112].

There is also a growing appreciation that antimicrobial peptides and certain antibiotics, such as daptomycin and colistin, share mechanistic similarities: both bind to and integrate into bacterial membranes, causing depolarization, disruption of ion gradients, and loss of membrane integrity, ultimately leading to bacterial death [107]. By disrupting membranes, including the Gram-negative outer membrane, antimicrobial peptides can increase bacterial susceptibility to antibiotics that might otherwise be ineffective. For instance, peptides like LL-37 enable vancomycin, traditionally ineffective against Gram-negative bacteria, to cross the outer membrane and reach its peptidoglycan target, where it can exert bactericidal effects [113,114]. Importantly, ESBL-producing *E. coli* often exhibit collateral sensitivity to azithromycin and colistin [115]. β-lactamase expression can alter bacterial physiology, contributing to cell envelope stress and increased outer membrane permeability that heighten susceptibility to these antibiotics, suggesting that β-lactamase expression may also increase susceptibility to cationic antimicrobial peptides such as cathelicidin [116]. These effects highlight the broader potential for antibiotics to synergize with host immune factors and improve therapeutic outcomes in certain infections.

Standard antimicrobial susceptibility testing, typically performed in bacteriological media devoid of host factors, does not capture these interactions. In the face of rising antibiotic resistance, understanding how antibiotics interact with innate immunity could expand therapeutic possibilities and inform the design of new antimicrobial agents that leverage such synergy.

## 8. Selected Experimentation

### 8.1. Experimental Rationale and Hypothesis

Given existing evidence that cathelicidin can potentiate the activity of diverse antibiotics and increase bacterial susceptibility, we hypothesized that the discordance between in vitro resistance and clinical response in some pediatric UTIs may reflect an in vivo interaction between antibiotic and cathelicidin, an effect not captured by standard susceptibility testing. To investigate the role of host immune interactions in the treatment of ESBL-producing *E. coli* UTIs, we evaluated the potential synergies between the antimicrobial peptide LL-37 and cephalexin, a commonly prescribed empiric therapy for pediatric UTIs, against clinical ESBL-UPEC isolates. This investigation was motivated by clinical observations of apparent cure following discordant antibiotic therapy, as well as by growing evidence that innate immunity can augment antibiotic activity in vivo.

**Figure 1:**
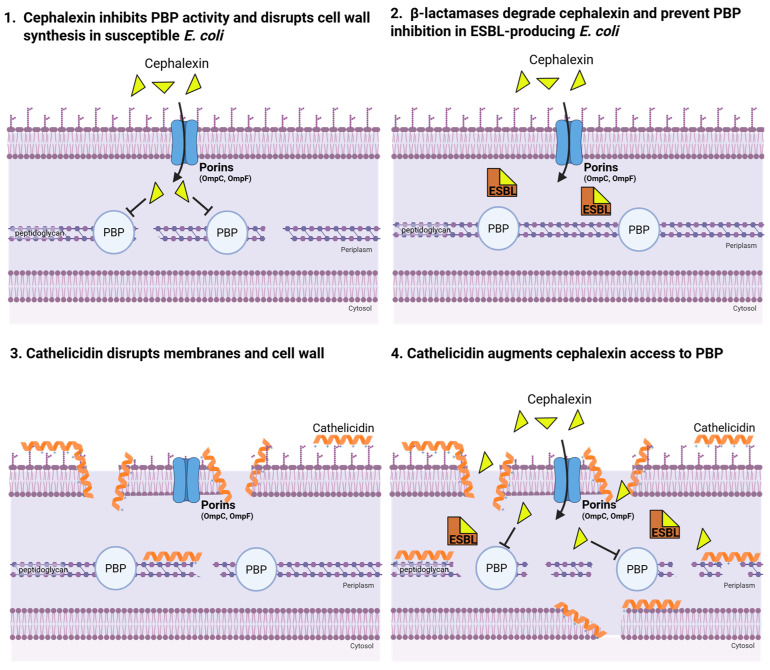
Mechanism of action for cephalexin and cathelicidin. (**1**) Cephalexin enters the periplasm via non-specific porins to inhibit cell wall synthesis by irreversibly binding to penicillin-binding proteins (PBPs), which catalyzed peptidoglycan synthesis. (**2**) In ESBL-producing *E. coli*, β-lactamases, concentrated in the periplasm, cleave the β-lactam ring of cephalexin and prevent PBP inhibition. (**3**) The strong positive net charge of cathelicidin promotes binding to negatively charged outer membrane and bacterial proteins (LPS) and subsequent disruption of the outer membrane, cell wall and inner membrane [13,14]. (**4**) Potential synergy between cathelicidin and cephalexin in ESBL-producing UPEC infections: cathelicidin increases cephalexin access to target. Created in BioRender. Coady, A. (2025) https://BioRender.com/dtjxmhj (accessed on 25 September 2025).

We hypothesized that cathelicidin could synergize with cephalexin in several ways (Figure 1): (1) cephalexin-induced perturbation of the bacterial outer membrane may increase access to the periplasm and peptidoglycan target, sensitizing strains to lower antibiotic concentrations, and/or that (2) subinhibitory cephalexin exposure could induce morphological or phenotypic changes that heighten susceptibility to cathelicidin. We tested these potential interactions in vitro using a checkerboard MIC assay and in vivo with a murine model of UTI.

### 8.2. Materials and Methods

Bacterial strains and growth conditions. *E. coli* strains used in these experiments include the virulent, non-ESBL-producing UPEC strain UTI89 [117], and clinical strains of ESBL-producing UPEC obtained from children diagnosed with UTIs at Rady Children’s Hospital San Diego (UCSD IRB 200120). Cultures were grown overnight in Luria broth (LB; Hardy Diagnostics) and preserved in 40% glycerol at −80 °C. For experimental use, frozen stocks were struck weekly onto Luria agar (LA) plates to obtain single colonies. For MIC and checkerboard assays, single colonies were grown overnight at 37 °C with shaking. Overnight cultures were diluted 1:100 in fresh media, and incubated to an OD600 0.4. For murine experiments, bacteria were grown stationary overnight in LB at 37 °C, washed once in sterile Dulbecco’s phosphate-buffered saline (D-PBS), and diluted to a final concentration of 1 × 10^7^ CFU/mL.

Minimum inhibitory concentration testing. Minimum inhibitory concentrations (MICs) for cephalexin and LL-37 (American Peptide) were determined for eight ESBL-producing UPEC isolates following Clinical and Laboratory Standards Institute (CLSI) guidelines [118]. Cephalexin MIC testing was performed in three growth media: cation-adjusted Mueller–Hinton Broth (CA-MHB), Roswell Park Memorial Institute 1640 supplemented with 10% Luria Broth (RPMI + 10% LB), and synthetic urine (SU) [119]. LL-37 MICs were assessed in RPMI + 10% LB. MIC results were determined based on visual turbidity and absorbance (OD600) after incubation at 37 °C for 15 h.

Checkboard assay. Checkerboard assays were performed in RPMI + 10% LB as previously described [120]. Fractional inhibitory concentration indices (FICIs) were interpreted as follows: synergy, FICI of ≤0.50; additivity, FICI of >0.50 to ≤1.0; no interaction (indifference), FICI of >1 to ≤4; antagonism, FICI of >4 [121].

Murine infection. Animal experiments were approved by the University of California San Diego (UCSD) Institutional Animal Care and Use Committee, and were performed under veterinary supervision. C57Bl/6J mice (strain 000664), originally purchased from Jackson Laboratories, were bred and maintained in-house. Mice were allowed to eat and drink ad libitum. Female mice aged 8–10 weeks were anesthetized with isoflurane and fifty microliters of prepared bacterial suspension (1 × 10^7^ CFU/mL) was introduced into the bladders via transurethral catheterization [122]. Beginning 24 h post-infection, mice were treated twice daily via oral gavage with water (control) or cephalexin (200 mg/kg). On day 4, mice were humanely euthanized, and kidneys and bladders harvested. For quantification of bacterial burden, tissues were homogenized in PBS containing 1 mm zirconia-silica beads (BioSpec Products, Inc., Bartlesville, OK, USA) using a MagNA Lyser (Roche, Basel, Switzerland), serially diluted, and plated on LA.

### 8.3. Results

#### 8.3.1. In Vitro Activities of Cephalexin and LL-37 Against ESBL UPEC

The clinical course of patients treated during UTI treatment with discordant antibiotics is summarized in Table 2, with full susceptibility results in Table 3. Minimum inhibitory concentrations (MICs) for cephalexin and LL-37 (American Peptide) were determined for eight ESBL-producing UPEC isolates following Clinical and Laboratory Standards Institute (CLSI) guidelines [119] using standard media (CA-MHB) as well as RPMI + 10% LB and SU. RPMI and SU were included due to growing evidence that they better mimic in vivo host environments compared to standard bacteriological media [111,123,124]. Growth of the isolates in SU was inconsistent, with failure to reach mid-log phase growth by 6 h, prompting the discontinuation of further testing in that medium. All eight isolates were uniformly resistant to cephalexin (MIC >16 mg/dL) across all tested media, while LL-37 MICs ranged from 4–16 µM (Table 2). When tested in combination via checkerboard assay, cephalexin and LL-37 showed an indifferent interaction (FICI >1 but ≤4) in all strains under both media conditions (Table 2).

#### 8.3.2. Cephalexin Treatment Fails to Reduce Bacterial Burden in Murine UTI Model

**Figure 2:**
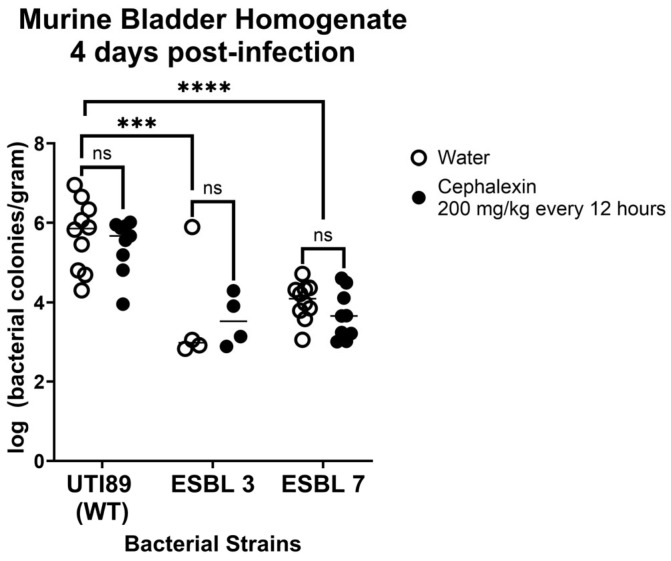
Bacterial Burden in the Bladder of Mice Inoculated with *E. coli* Strains Following Cephalexin Treatment. Female C57Bl/6J mice were infected with bacteria via transurethral catheterization. Beginning 24 h post-infection, mice were treated twice daily via oral gavage with water (control) or cephalexin (200 mg/kg). On day 4, to assess early response to treatment, mice were humanely euthanized, and their kidneys and bladders harvested for quantitation of bacterial burden. A two-way ANOVA with Sidak’s multiple comparison test correction was performed using GraphPad Prism, v8.1.2 (GraphPad Software Inc., La Jolla, CA, USA) and *p* < 0.05 was considered statistically significant. *** *p* < 0.001, **** *p* < 0.0001, ns = non-significant.

Although we did not observe an effect of cathelicidin on cephalexin susceptibility, this does not exclude the possibility that cephalexin efficacy may be augmented in vivo by other host factors. To evaluate the potential contribution of host factors in enhancing antibiotic efficacy in vivo, we used a murine UTI model to assess the impact of cephalexin on bacterial burden [125]. This model is particularly suitable, as both humans and mice possess a single cathelicidin gene—LL-37 in humans and CRAMP in mice—which are structurally and functionally similar [40]. Two ESBL-producing clinical UPEC isolates (3 and 7) were selected for testing based on favorable response in patients treated with discordant therapy as indicated by sterile follow-up cultures. A wild-type, non-ESBL producing strain (*E. coli* UTI89) was included for comparison. Cephalexin is rapidly excreted after oral administration, with >80% of the drug detected in murine urine within 8 h after administration [126]. The recommended pediatric dosing for uncomplicated UTIs is 25–50 mg/kg/day, typically administered two to four times daily [127]. To approximate an equivalent human dose in our murine infection model, we adjusted for body surface area differences and administered cephalexin at 200 mg/kg twice daily (totaling 400 mg/kg/day) [128,129,130]. Mice received three consecutive days of treatment and were euthanized on day 4 post-infection to capture early treatment responses before substantial spontaneous bacterial clearance or late-stage inflammatory sequelae could confound interpretation. No bacteria were recovered from the kidneys in any group, suggesting that the infection was localized to the bladder. At day 4 post infection, cephalexin had no significant effect on bacterial burden in the bladder relative to control in any of the tested strains (Figure 2).

## 9. Discussion

This study explored the interaction between the innate immune peptide cathelicidin (LL-37) and cephalexin against pediatric clinical ESBL UPEC isolates. No synergy was observed between LL-37 and cephalexin in vitro, and cephalexin had no significant effect on bacterial burden in the bladder relative to control in any of the tested strains. This may reflect non-complementary mechanisms of action between cephalexin and LL-37. The strains may already be maximally susceptible to LL-37 with no additional benefit gained from cephalexin exposure. Alternatively, LL-37 may not interfere with the β-lactamase-mediated hydrolysis of cephalexin, allowing resistance to persist. However, our findings support the hypothesis that host factors and physiological environments influence bacterial fitness. Specifically, ESBL-producing strains exhibited impaired growth in synthetic urine, and lower bacterial burden in our murine UTI model compared to the non-ESBL UTI89 strain. These findings raise the possibility that certain resistant mechanisms, such as ESBL production, may confer a fitness cost in the urinary tract environment.

Interestingly, the murine model did not replicate the clinical benefit of cephalexin some pediatric patients with ESBL *E. coli* UTIs, suggesting that additional factors beyond cathelicidin or cephalexin pharmacodynamics may drive infection resolution in vivo. Cephalexin failed to reduce bacterial burden even in mice infected with the susceptible UTI89 strain, likely due to cephalexin’s poor epithelial penetration and inability to target *E. coli* within IBCs [131,132]. Although IBC formation by the ESBL strains was not confirmed in this study, this may explain the lack of clearance across in all strains after 4 days of therapy. The lower bladder colonization by ESBL-producing strains compared to UTI89 further suggest that reduced virulence or impaired IBC formation may underlie differences in pathogenicity. However, further studies are needed to test whether ESBL-producing strains exhibit reduced fitness or capacity to evade host defenses.

This study was distinctive in several key aspects. First, the clinical strains assessed were obtained directly from documented infections with associated clinical outcomes. Second, these isolates were evaluated under multiple physiologically relevant conditions to better recapitulate antibiotic susceptibility in vivo, including exposure to host factors, specifically cathelicidin, that may potentiate antibiotic efficacy. Finally, to our knowledge, this is the first study to employ a murine model using clinical isolates in which infection resolution was observed despite discordant in vitro antibiotic susceptibility. Although host immune interactions were not directly characterized, we did observe a significant difference in overall bacterial burden.

While our study provides valuable insights, there are several limitations to consider. The most significant limitation is the small overall sample size, as only a limited number of isolates were tested, which restricts the generalizability of the findings. In addition, we did not characterize the specific genetic determinants or plasmid vectors conferring ESBL resistance in these strains; therefore, mechanistic interactions between the resistance elements and cathelicidin cannot be specifically inferred. In addition, the in vitro and murine models used may not fully capture the complexity of human UTI pathogenesis, particularly with respect to immune maturation and urinary tract anatomy. Additionally, the synthetic urine used in the in vitro testing may not capture the full range of physiochemical variability present in human urine. The focus on cathelicidin (LL-37) as a primary immune effector leaves other potentially relevant factors—such as neutrophil extracellular traps, complement, and cytokine signaling—unexplored. Finally, mechanisms of resistance beyond ESBL production, such as biofilm formation or efflux pump activity, were not directly examined, but may also modulate therapeutic response.

## 10. Conclusions

Community-acquired UTIs caused by ESBL-producing bacteria are increasingly common in pediatric populations, presenting an emerging public health challenge. Remarkably, despite in vitro resistance to first-generation cephalosporins, some patients respond clinically to treatments like cephalexin. These observations suggest that host immune factors and environmental conditions may enhance antibiotic efficacy, highlighting the potential for alternative therapeutic strategies.

As antibiotic resistance continues to escalate, a better understanding of host–pathogen–drug interaction will be essential for guiding treatment and informing novel antimicrobial strategies. Future research should incorporate expanded mechanistic studies on fitness tradeoffs in ESBL strains, explore immune-based adjunct therapies, and refine experimental models to better recapitulate pediatric physiology. Integrating microbiology with host immunology may yield new insights for clinical management of drug-resistant UTIs and support broader efforts to preserve the utility of existing antibiotics.

## Figures and Tables

**Table 1:** Studies Demonstrating Favorable Outcomes Despite Discordant Antibiotics.

Study	Type	N	Male %	Age, Median, IQR, Months	*E. coli* %	Discordant Antibiotics, *n*, %	Anatomic Abnormalities, *n*, %	Clinical Improvement in 48 h, *n*, %
Katsuta et al. 2013 [29]	Retrospective	54	30%	28 months (92)	100%	32, 59.2%	20, 37.0%	24/32, 75%
Tratselas et al. 2011 [30]	Matched case–control	28	20%	2.25 (0.6–108)	67.9%	24, 85.7%	24, 85.7%	17/18, 94.4%
Toubiana et al. 2016 [31]	Retrospective	82	53%	1 (0.72–192)	100%	51, 62.2%	44, 48.9%	
Madhi et al. 2018 [32]	Prospective	301	44.5%	12 (0.02–17.9)	87.8%		67, 22.3% **	
Hyun et al. 2019 [33]	Retrospective	146	63%	7.2 (0–24)	80.1%	109, 74.7%	43, 29.5%	97/109, 88.9%
Wang et al. 2020 [34]	Retrospective	316	22%	28.8 (7.2–78.0)	98.7%	316, 100%	16, 5.1% ^††^	192/230, 83.4%
Tamas et al. 2022 [35]	Retrospective	45	37.8%	5.42	93%	41, 91.1%	14, 31.1%	

** Includes VUR in addition to anatomic abnormalities. ^††^ History of VUR or renal abnormalities

**Table 2:** Clinical Data and In Vitro Studies of Cephalexin and LL-37 Against ESBL *Escherichia coli* UTI Strains.

Strain Number	Patient Age/Sex	Clinical Course ^1^	Cephalexin		LL-37	Cephalexin + LL-37
			MIC (mg/L)		MIC (µM)	FICI ^2^
			CA-MHB	RPMI + 10% LB	RPMI + 10% LB	RPMI + 10% LB
3	17 yo, F	Improved	>256	>256	8	2
6	17 yo, F	ND	>256	>256	16	2
7	1 yo, F	Improved	>256	>256	8	2
9	8 yo, F	ND	>256	>256	8	2
15	4 yo, F	ND	>256	>256	8	2
17	4 yo, F	ND	>256	>256	4	2
18	16 yo, F	ND	>256	>256	4	2
21	20 do, M	ND	>256	>256	8	2

^1^ Clinical Course indicates whether clinical improvement was observed while on discordant antibiotics. ‘Improved’ is defined as a repeat urine culture showing clearance of the pathogen, while ‘ND’ (not determined) refers to cases where no repeat culture was performed. ^2^ FICIs were interpreted as follows: synergy, FICI of ≤0.50; additivity, FICI of >0.50 to ≤1.0; no interaction (indifference), FICI of >1 to ≤4; antagonism, FICI of >4 [121]. Abbreviations: ESBL, extended spectrum β-lactamases; UTI, urinary tract infection; MIC, minimum inhibitory concentration; FICI, fractional inhibitory concentration index; CA-MHB, cation-adjusted Mueller–Hinton Broth; RPMI + 10% LB, Roswell Park Memorial Institute 1640 supplemented with 10% Luria Broth; yo, year-old; do, day-old; F, female; M, male.

**Table 3:** Antibiotic Susceptibility Interpretations for ESBL *Escherichia coli* Strains from UTIs.

	Strain Number							
Antibiotic	3	6	7	9	15	17	18	21
Cephalexin	R	R	R	R	R	R	R	R
Amikacin	S	S	S	S	S	S	S	S
Ciprofloxacin	S	S	ND	ND	ND	ND	ND	S
Meropenem	S	S	S	S	S	S	S	S
Piperacillin-tazobactam	S	S	S	S	S	S	S	S
Nitrofurantoin	S	S	S	S	S	S	S	S
Tobramycin	I	S	S	ND	ND	ND	S	S
Trimethoprim-sulfamethoxazole	S	S	ND	S	S	S	ND	ND
Gentamicin	ND	S	S	ND	ND	ND	S	S

Abbreviations: ESBL, extended spectrum β-lactamases; S, susceptible; I, intermediate; ND, no data.

## Data Availability

All data generated or analyzed during this study are included in this published article. Additional data are available from the corresponding author on reasonable request.

## Abbreviations

The following abbreviations are used in this manuscript:

UTI	Urinary tract infection
UPEC	Uropathogenic *Escherichia coli*
ESBL	Extended-spectrum β-lactamases
LL-37	Human cathelicidin
ED	Emergency departments
IBCs	Intracellular bacterial communities
AMPs	Antimicrobial peptides
CAKUT	Congenital abnormalities of the kidney and urinary tract
VUR	Vesicourethral reflex
AAP	American Academy of Pediatrics
TMP-SMX	Trimethoprim-sulfamethoxazole
IDSA	Infectious Diseases Society of America
ESBL-E	ESBL-Enterobacterales
PBPs	Penicillin binding proteins
MRSA	Methicillin-resistant *Staphylococcus aureus*
CLSI	Clinical and Laboratory Standards Institute
MIC	Minimum inhibitory concentration
LB	Luria broth
LA	Luria agar
CA-MHB	Cation-adjusted Mueller–Hinton Broth
RPMI	Roswell Park Memorial Institute 1640
D-PBS	Dulbecco’s Phosphate-Buffered Saline
SU	Synthetic urine
FICI	Fractional inhibitory concentration index
